# Role of NADPH Oxidase-Derived ROS-Mediated IL-6/STAT3 and MAPK/NF-κB Signaling Pathways in Protective Effect of Corilagin against Acetaminophen-Induced Liver Injury in Mice

**DOI:** 10.3390/biology12020334

**Published:** 2023-02-20

**Authors:** Fu-Chao Liu, Hung-Chen Lee, Chia-Chih Liao, An-Hsun Chou, Huang-Ping Yu

**Affiliations:** 1 Department of Anesthesiology, Chang Gung Memorial Hospital, Linkou Branch, Taoyuan 333, Taiwan; 2 College of Medicine, Chang Gung University, Taoyuan 333, Taiwan

**Keywords:** acetaminophen, corilagin, inflammation, liver injury, NOX, NF-κB, ROS, STAT3

## Abstract

**Simple Summary:**

Acetaminophen overdose causes acute liver injury by excessive oxidative stress. The present study examines the mechanisms underlying the protective effect of corilagin against acetaminophen-induced liver injury. Our results showed that corilagin attenuates the liver injury through its anti-oxidant and anti-inflammatory properties. Corilagin may be a therapeutic agent for acute liver injury.

**Abstract:**

Acetaminophen (APAP) overdose causes acute liver injury via oxidative stress, uncontrolled inflammatory response, and subsequent hepatocyte death. Nicotinamide adenine dinucleotide phosphate oxidase (NOX) is a potent source of cellular reactive oxygen species (ROS) and may contribute to oxidative stress in many inflammatory processes. Corilagin, a component of *Phyllanthus urinaria*, possesses antioxidant, anti-inflammatory, and hepatoprotective effects. We evaluated the mechanisms underlying the protective effect of corilagin against acetaminophen-induced liver injury. Mice were intraperitoneally administrated 300 mg/kg APAP or equal volume of saline (control), with or without various concentrations of corilagin (0, 1, 5, or 10 mg/kg) administered after 30 min. All animals were sacrificed 16 h after APAP administration, and serum and liver tissue assays including histology, immunohistochemistry, and Western blot assay were performed. Corilagin post-treatment significantly attenuated APAP-induced liver injury (*p* < 0.005), inflammatory cell infiltration, hepatic proinflammatory cytokine levels, and hepatic oxidative stress. Furthermore, corilagin attenuated the protein levels of NOX1, NOX2, signal transducer and activator of transcription 3 (STAT3), and nuclear factor kappa B (NF-κB) in APAP-induced liver injury. These results indicated that the antioxidant, anti-inflammatory, and protective effects of corilagin in APAP-induced liver injury might involve the regulation of interleukin (IL)-6/STAT3 and mitogen-activated protein kinase (MAPK)/NF-κB signaling pathways through NOX-derived ROS.

## 1. Introduction

Acetaminophen, *N*-acetyl-*p*-aminophenol (APAP), is commonly used for antipyretics and analgesics globally [1]. APAP is considered safe at proper therapeutic doses; however, accidental or unintentional overdose may lead to acute liver injury [2,3]. APAP is metabolized to its inactive glucuronide and sulfate conjugates in liver [4]. Only a small amount of APAP is oxidized to the metabolite *N*-acetyl-*p*-benzoquinone imine (NAPQI), which is inactivated to form non-toxic compounds [5,6]. Excessive NAPQI formation can result in oxidative stress and uncontrolled inflammatory response [3,7,8].

Oxidative stress is critical for APAP-induced organ injury [9,10]. Briefly, protein adducts formed through the reactive intermediate NAPQI augment mitochondrial reactive oxygen species (ROS) production [11,12]. Excess ROS production can promote mitogen-activated protein kinases (MAPKs) including c-Jun N-terminal kinase (JNK) and extracellular signal-regulated kinase (ERK) [13,14]. Previous studies demonstrated that JNK activation could also amplify ROS formation [15]. Moreover, sustained activation of MAPK was shown to contribute to inflammatory response and cell death [16,17].

Corilagin, an important major component of *Phyllanthus urinaria*, possesses antioxidant and hepatoprotective effects [18,19,20]. In sepsis, corilagin was shown to ameliorate an inflammatory response through the toll-like receptor 4 (TLR4) [21]. In a recent study, corilagin protected against cerebral ischemic injury [22]. In hemorrhagic shock, corilagin was hepatoprotective [23]. Furthermore, we recently demonstrated that corilagin was protective against acute lung injury [24]. We also demonstrated that corilagin was protective through the suppression of inflammation; however, mechanisms underlying the antioxidant and anti-inflammatory roles of corilagin were unclear [25]. Therefore, we aimed to elucidate these mechanisms in the present study.

## 2. Methods

### 2.1. Mice

Adult male C57BL/6C mice were used. All experimental procedures were approved by Chang Gung Memorial Hospital.

### 2.2. Animal Model

Mice were assigned to one of six groups, in which acetaminophen was dissolved. In four of the six groups, mice received 300 mg/kg acetaminophen, followed by intraperitoneal administration of corilagin (Sigma Chemical, St. Louis, MO, USA) (0, 1, 5, or 10 mg/kg). In the remaining two groups, mice were intraperitoneally injected with normal saline, then the intraperitoneal was injected with phosphate-buffered saline or corilagin (10 mg/kg). After 16 h, the animals were sacrificed and tissue and blood samples were collected.

### 2.3. Serum Alanine Transaminase (ALT) and Aspartate Transaminase (AST) Measurement

Serum ALT and AST concentrations were measured and blood samples were collected. Then, the collected supernatants were analyzed.

### 2.4. Histology and Immunohistochemistry

At the end of the experiments, left liver lobes were harvested and fixed in 4% paraformaldehyde. For histological examination, the samples were stained with hematoxylin and eosin. For immunohistochemistry, the samples were incubated with antibodies for the neutrophil marker Ly6G, the macrophage marker Mac-2, or nicotinamide adenine dinucleotide phosphate oxidase (NOX) 2.

### 2.5. Interleukin (IL)-6 and Tumor Necrosis Factor (TNF)-α Levels

Liver tissues were homogenized. The supernatants were used to measure IL-6 and TNF-α concentrations with a spectrometer (absorbance of 450 nm) using ELISA Kits (R&D Systems Minneapolis, MN, USA).

### 2.6. Myeloperoxidase (MPO) Activity, Malondialdehyde (MDA), and Glutathione (GSH) in Liver

Supernatants were used to measure malondialdehyde levels and to determine glutathione levels. For myeloperoxidase activity, liver tissues were homogenized. Absorbance of the samples was measured over 5 min.

### 2.7. Western Blot

Membranes were incubated with antibodies to NOX1, NOX2, IL-6, ERK, phospho-ERK, JNK, phospho-JNK, signal transducer and activator of transcription 3 (STAT3), and nuclear factor kappa B (NF-κB).

### 2.8. Statistical Analysis

Data were presented as means ± standard error of the mean. One-way analysis of variance and Tukey’s multiple comparison test were used to analyze the results. A *p*-value of <0.05 was considered as statistically significant.

## 3. Results

### 3.1. Effects of Corilagin on APAP-Induced Liver Injury

**Figure 1:**
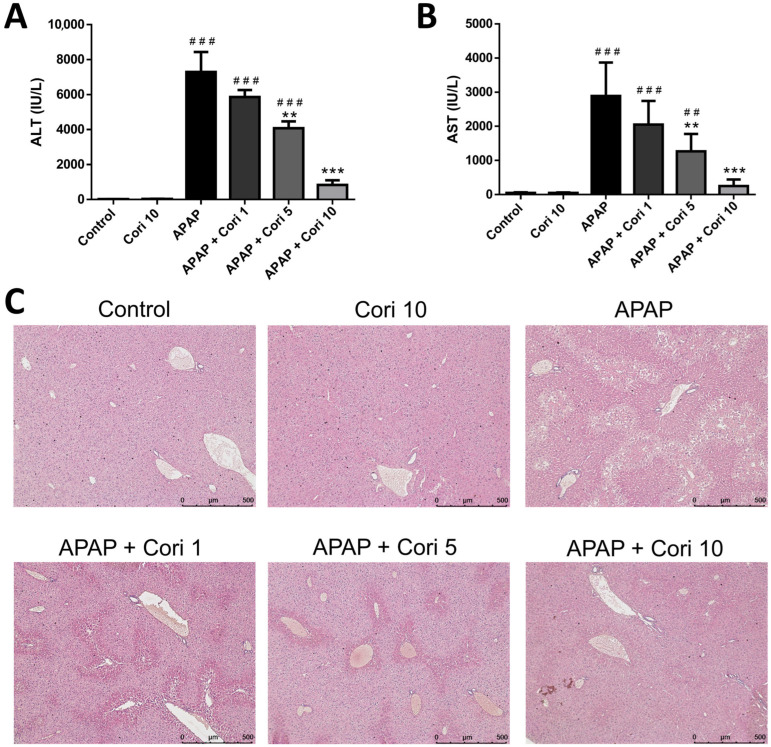
The effects of corilagin on serum ALT levels (**A**), AST levels (**B**), and histopathological changes (**C**) in acetaminophen-induced hepatic injury. Mice were injected with APAP (300 mg/kg) or equal volume of saline (control), and treated with corilagin (0, 1, 5, or 10 g/kg) after 30 min. Each value represents mean ± SEM; n = 6 for each group. ^##^ *p* < 0.01, ^###^ *p* < 0.005 vs. control group; ** *p* < 0.01, *** *p* < 0.005 vs. APAP group (50×).

ALT and AST levels were increased in the APAP group (*p* < 0.005), whereas no difference was observed between the control and 10 mg/kg corilagin groups. Treatment with 5 and 10 mg/kg corilagin significantly reduced serum ALT and AST levels. However, serum ALT and AST concentrations were not decreased significantly following the treatment with 1 mg/kg corilagin (Figure 1A,B).

Histological examination demonstrated severe centrilobular hepatic necrosis in the APAP group. Clearly, the treatment with 5 and 10 mg/kg corilagin attenuated the pathological changes (Figure 1C).

### 3.2. Effects of Corilagin on Hepatic Accumulation of Neutrophils and Macrophages in APAP-Induced Hepatic Injury

**Figure 2:**
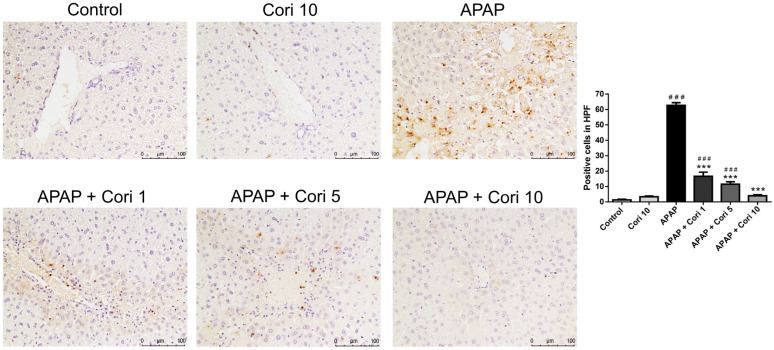
The effects of corilagin on neutrophil infiltration in acetaminophen-induced hepatic injury. Animals were injected with APAP (300 mg/kg) or equal volume of saline (control), and treated with corilagin (0, 1, 5, or 10 g/kg) (200×). Each value represents mean ± SEM; n = 6 for each group. ^###^ *p* < 0.005 vs. control group; *** *p* < 0.005 vs. APAP group.

Immunohistochemistry of liver tissue sections using an antibody to Ly6G, a granulocyte-specific marker, revealed overt neutrophil infiltration of the necrotic areas in the APAP group (Figure 2). The groups treated with corilagin following APAP administration exhibited significantly decreased neutrophil infiltration in liver parenchyma.

**Figure 3:**
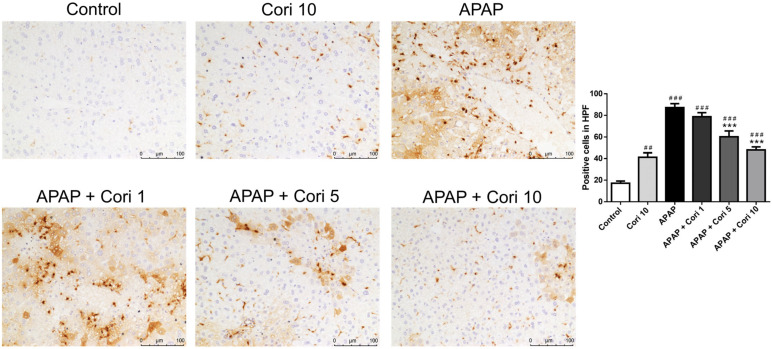
The effects of corilagin on macrophage infiltration in acetaminophen-induced hepatic injury. Animals were injected with APAP (300 mg/kg) or equal volume of saline (control), and treated with corilagin (0, 1, 5, or 10 g/kg) (200×). Each value represents mean ± SEM; n = 6 for each group. ^##^ *p* < 0.01, ^###^ *p* < 0.005 vs. control group; *** *p* < 0.005 vs. APAP group.

To evaluate macrophage infiltration following APAP-induced injury, the liver tissue sections were immunohistochemically stained using an antibody to Mac-2. The APAP group exhibited increased macrophage infiltration of the necrotic areas, whereas the groups treated with corilagin following APAP administration exhibited significantly decreased macrophage infiltration in liver parenchyma (Figure 3).

### 3.3. Effects of Corilagin on Hepatic IL-6 and TNF-α Levels

**Figure 4:**
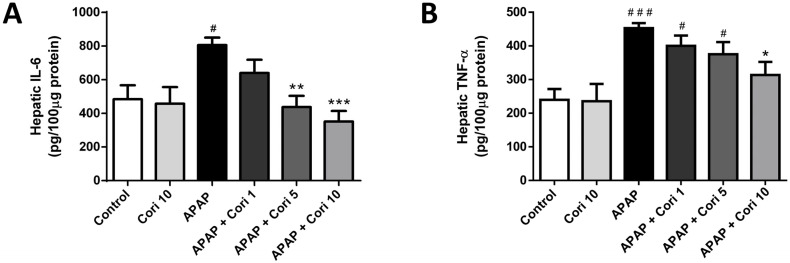
The effects of corilagin on IL-6 (**A**) and TNF-α (**B**) concentrations in acetaminophen-induced hepatic injury. Animals were intraperitoneally injected with APAP (300 mg/kg) or equal volume of saline (control), and administrated with various concentrations of corilagin (0, 1, 5, or 10 g/kg) after 30 min. ^#^ *p* < 0.05, ^###^ *p* < 0.005 vs. control group; * *p* < 0.05, ** *p* < 0.01, *** *p* < 0.005 vs. APAP group.

Hepatic IL-6 and TNF-α levels, which were increased in the APAP group, did not differ between the control and 10 mg/kg corilagin alone groups (Figure 4A). Importantly, treatment with 5 mg/kg corilagin significantly reduced hepatic IL-6 levels (*p* < 0.01). Moreover, IL-6 and TNF-α were lower in the group treated with 10 mg/kg corilagin following APAP administration (Figure 4B).

### 3.4. Corilagin on Hepatic MDA and GSH Levels and MPO Activity

**Figure 5:**
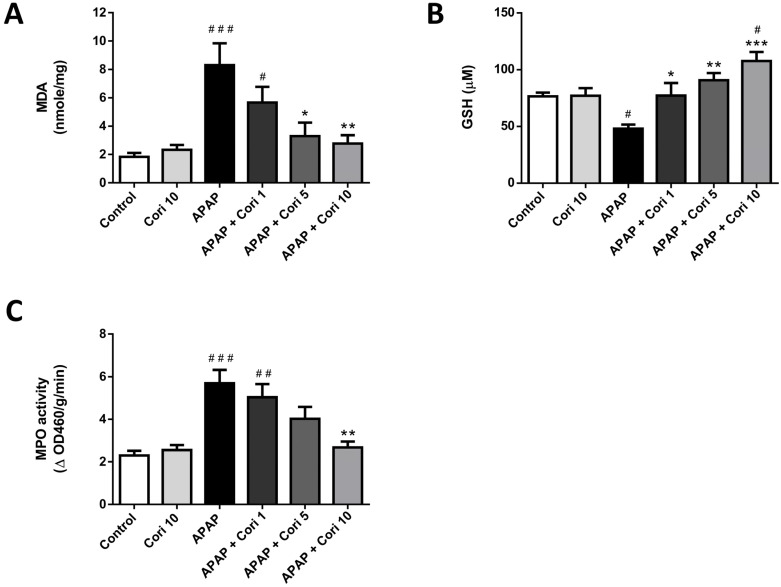
The effects of corilagin on MDA (**A**), GSH (**B**) levels, and MPO activity (**C**) in acetaminophen-induced hepatic injury. Animals were injected with APAP (300 mg/kg) or equal volume of saline (control), and treated with corilagin (0, 1, 5, or 10 g/kg). ^#^ *p* < 0.05, ^##^ *p* < 0.01, ^###^ *p* < 0.005 vs. control group; * *p* < 0.05, ** *p* < 0.01, *** *p* < 0.005 vs. APAP group.

In Figure 5, MDA, a lipid peroxidation marker, which was markedly increased in the APAP group, was reduced after the treatment with 5 and 10 mg/kg corilagin. Moreover, hepatic levels of GSH, a cellular antioxidant marker, which was significantly lower in the APAP group, was significantly higher after the treatment with 1, 5, and 10 mg/kg corilagin. Finally, MPO activity, used as a marker for neutrophil infiltration and oxidant levels, which was increased in the APAP group, was decreased after the treatment with corilagin.

### 3.5. Effects of Corilagin on Hepatic NOX1 and NOX2 Expressions

**Figure 6:**
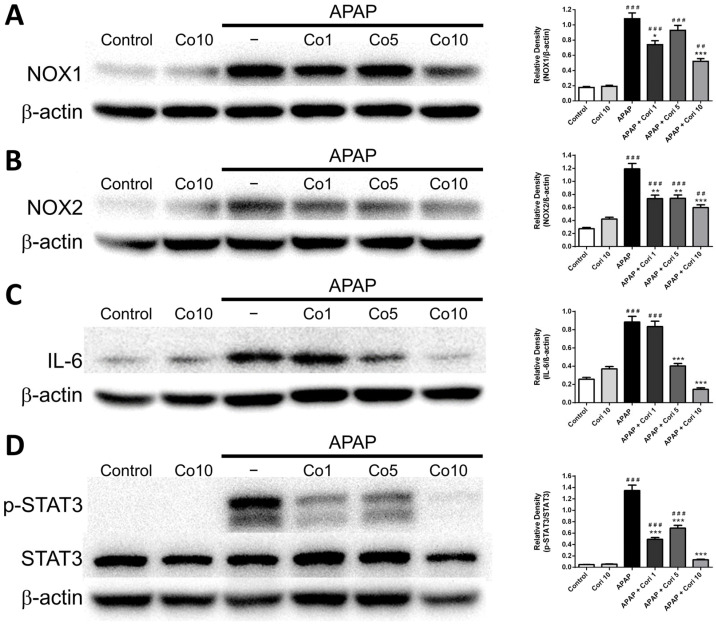
The effects of corilagin on NOX1 (**A**), NOX2 (**B**), IL-6 (**C**), and STAT3 (**D**) expression. Animals were injected with APAP (300 mg/kg) or equal volume of saline (control), and treated with corilagin (0, 1, 5, or 10 g/kg) after 30 min. ^##^ *p* < 0.01, ^###^ *p* < 0.005 vs. control group; * *p* < 0.05, ** *p* < 0.01, *** *p* < 0.005 vs. APAP group. Figures S1–S4 Uncropped Western blots.

**Figure 7:**
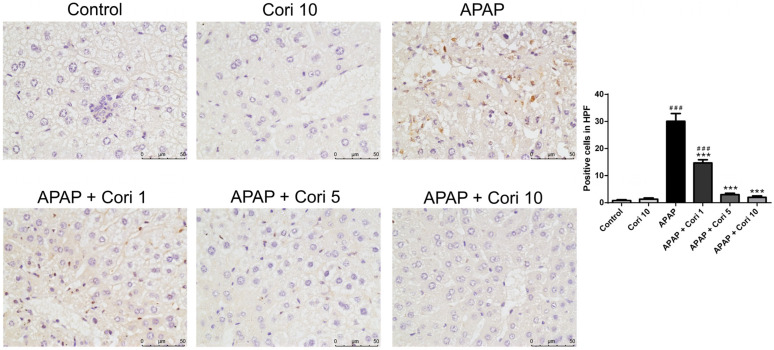
The effects of corilagin on NOX2 expression. Animals were injected with APAP (300 mg/kg) or equal volume of saline (control), and treated with corilagin (0, 1, 5, or 10 g/kg) (400×). Each value represents mean ± SEM; n = 6 for each group. ^###^ *p* < 0.005 vs. control group; *** *p* < 0.005 vs. APAP group.

We measured NOX1 and NOX2 levels in liver tissue using Western blotting. Hepatic NOX1 and NOX2 levels, which were comparable between the control and 10 mg/kg corilagin alone groups, were higher in the APAP group (Figure 6A,B). Notably, the treatment with corilagin significantly reduced hepatic NOX1 and NOX2 levels (*p* < 0.005 for both). Furthermore, immunohistochemical staining revealed increased hepatic NOX2 levels in the APAP group (Figure 7), which were clearly reduced after administration with 5 and 10 mg/kg corilagin following APAP administration (Figure 7).

### 3.6. Effects of Corilagin on IL-6 and STAT3 Concentrations

We measured hepatic IL-6 and STAT3 concentrations using Western blotting. Hepatic IL-6 and STAT3 levels, which were not different between the control and 10 mg/kg corilagin alone groups, were significantly higher in the APAP group (Figure 6C,D). Additionally, administration with corilagin following APAP administration significantly reduced hepatic IL-6 and STAT3 levels (*p* < 0.005 for both).

### 3.7. Hepatic NF-κB, Phospho-JNK, and Phospho-ERK Levels

**Figure 8:**
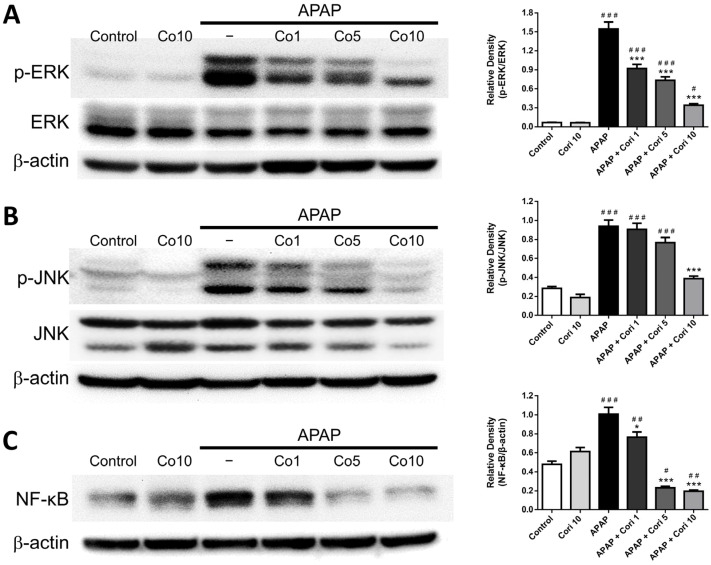
The effects of corilagin on ERK (**A**), JNK (**B**), and NF-kB (**C**) expression. Animals were injected with APAP (300 mg/kg) or equal volume of saline (control), and administered with corilagin (0, 1, 5, or 10 g/kg). ^#^ *p* < 0.05, ^##^ *p* < 0.01, ^###^ *p* < 0.005 vs. control group; * *p* < 0.05, *** *p* < 0.005 vs. APAP group. Figures S5–S7: Uncropped Western blots.

Finally, we determined changes in NF-κB, phospho-JNK, and phospho-ERK. NF-κB, phospho-JNK, and phospho-ERK were not significantly different between the control and 10 mg/kg corilagin alone groups. However, hepatic levels of all three parameters were increased in the APAP group (Figure 8A–C). Administration with 5 mg/kg corilagin significantly reduced hepatic phospho-ERK and NF-κB levels (Figure 8A,C). Furthermore, administration with corilagin significantly reduced hepatic phospho-ERK, phospho-JNK, and NF-κB levels (*p* < 0.005 for all) (Figure 8A–C).

## 4. Discussion

Our analyses revealed that corilagin post-treatment reduced APAP-induced hepatic injury, inflammation, and oxidation. Moreover, corilagin post-treatment significantly reduced hepatic NOX1, NOX2, IL-6, STAT3, phospho-ERK, phospho-JNK, and NF-κB levels. Altogether, these results show the protection of corilagin against APAP-induced hepatic injury and suggest that its antioxidant and anti-inflammatory effect occurs through NOX-derived ROS-mediated regulation of the IL-6/STAT3 and MAPK/NF-κB.

Innate immune system is important for APAP-induced hepatic injury [26,27,28]. Hepatocyte apoptosis or necrosis activate Kupffer cells through recognition by TLR [29,30,31]. Hepatic recruitment of monocytes and neutrophils secrete proinflammatory cytokines that contribute to an extensive inflammatory response and the subsequent severe liver damage [32,33,34,35]. Consistent with our previous work, we herein demonstrate that corilagin post-treatment ameliorates APAP-induced hepatic injury and reduces inflammatory cell infiltration and proinflammatory cytokine levels, confirming that corilagin exhibits protective effects for APAP-induced hepatic injury.

Progressive oxidative stress is characterized for APAP-induced hepatic injury [36,37]. Oxidative stress is a function of ROS overproduction in the setting of relative deficiency in antioxidant defenses [38,39]. Imbalanced ROS generation and consequent free radical production may result in injury [40]. Malondialdehyde is the main final product of lipid peroxidation in cells, and malondialdehyde levels frequently serve as an indicator of oxidative stress [41]. Myeloperoxidase represents the degree of neutrophil infiltration, and myeloperoxidase levels are a common biomarker of oxidative damage [42]. A recent study demonstrated that corilagin reduced oxidative stress in APAP-induced hepatic injury [43]. In agreement, we found that corilagin post-treatment led to reductions in malondialdehyde and myeloperoxidase levels, indicating reduced ROS production. Moreover, we found that corilagin post-treatment led to increased glutathione levels, indicating enhanced antioxidant capacity. Altogether, the results show that corilagin exerts protection against APAP-induced liver injury through antioxidant mechanisms.

A previous study demonstrated that excess ROS production mediated through NOX activation played a vital role in neutrophil-mediated inflammatory response and liver injury in a hemorrhagic shock model [44]. A recent study reported that NOX1-derived ROS played a critical role in liver disease [45]. Moreover, NOX2, primarily expressed in Kupffer cells, macrophages, and neutrophils, plays an important role in inflammation through ROS release in many inflammatory conditions [46]. Furthermore, we recently reported that corilagin post-treatment attenuated lipopolysaccharide-induced lung injury through the inhibition of NOX2 pathway [24]. We found that the marked increase in hepatic NOX1 and NOX2 levels was attenuated with corilagin post-treatment, suggesting that the antioxidant effect of corilagin was, at least partially, through the downregulation of NOX1 and NOX2 levels in liver.

STAT3, a transcription factor activated by several proinflammatory cytokines, such as IL-6, regulates diverse cellular functions, such as inflammation, survival, differentiation, and proliferation [47]. STAT3 signaling is associated with liver injury, fibrosis, inflammatory response, and oncogenesis [48]. Previous studies have shown the significant role of STAT3 in hepatic injury [49]. A recent study demonstrated that treatment with a STAT3 inhibitor attenuated acute liver injury by regulating macrophages and reducing proinflammatory cytokine levels [50]. Corilagin post-treatment reduced IL-6 and STAT3 levels, suggesting that the anti-inflammatory effect of corilagin might occur via the regulation of IL-6/STAT3.

MAPK family members, including ERK and JNK, play essential roles in differentiation, inflammation, and oxidative stress [51]. MAPK activation can lead to the induction of NF-κB pathway and subsequent increases in proinflammatory cytokine secretion and augmentation of inflammation [52]. Consistent with our previous work, we found that APAP challenge led to significant increases in phospho-JNK, phospho-ERK, and NF-κB, which were significantly reduced with corilagin post-treatment, suggesting that the anti-inflammation of corilagin was through MAPK signaling pathway.

## 5. Conclusions

In conclusion, corilagin was beneficial for APAP-induced hepatic injury through its antioxidation and anti-inflammation. The underlying mechanisms might involve the regulation of IL-6/STAT3 and MAPK/NF-κB mediated with NOX-derived ROS.

## Data Availability

The data presented in this study are available on request from the corresponding author.

## Supplementary Materials

The following supporting information can be downloaded at: https://www.mdpi.com/article/10.3390/biology12020334/s1, Figure S1: Uncropped Western blots from Figure 6A.; Figure S2: Uncropped Western blots from Figure 6B; Figure S3. Uncropped Western blots from Figure 6C; Figure S4. Uncropped Western blots from Figure 6D; Figure S5. Uncropped Western blots from Figure 8A. Figure S6. Uncropped Western blots from Figure 8B. Figure S7. Uncropped Western blots from Figure 8C.
