# Supplementary material for: Role of NADPH Oxidase-Derived ROS-Mediated IL-6/STAT3 and MAPK/NF-κB Signaling Pathways in Protective Effect of Corilagin against Acetaminophen-Induced Liver Injury in Mice
Source: Biology (Basel). 2023 Feb 20;12(2):334. doi: 10.3390/biology12020334 (PMC9952884; doi:10.3390/biology12020334)
Supplement: Supplementary file 1 [file biology-12-00334-s001.zip › biology-2196233-supplementary.pdf]

biology

Supplementary Materials:

Role of NADPH Oxidase-derived ROS-mediated IL-6/STAT3 and MAPK/NF-κB Signaling Pathways in Protective Effect of Corilagin against Acetaminophen-induced Liver Injury in Mice

Fu-Chao Liu [1,2,†], Hung-Chen Lee [1,2,†], Chia-Chih Liao [1,2], An-Hsun Chou [1,2] and Huang-Ping Yu [1,2,*]

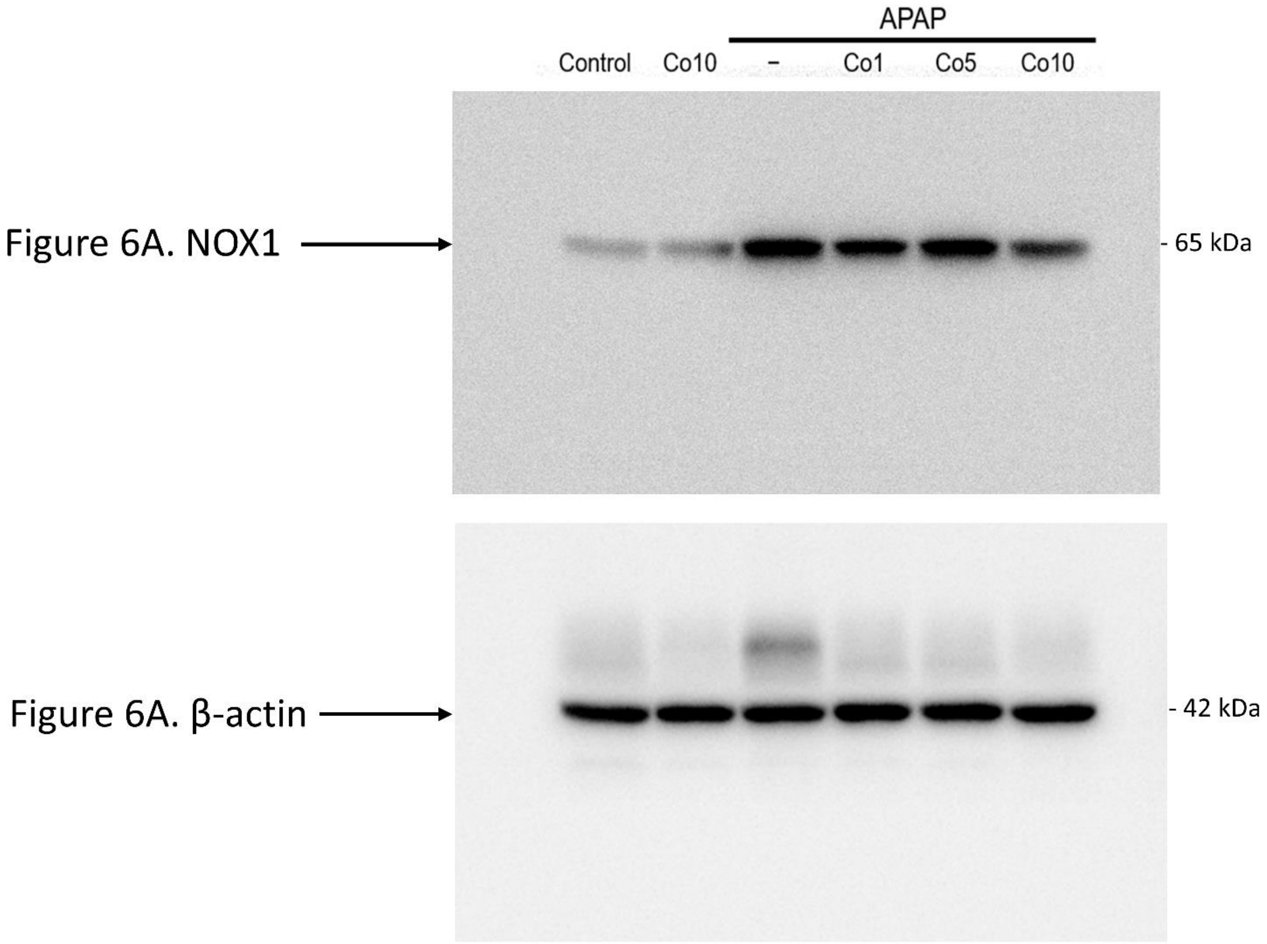

Figure S1. Uncropped Western blots from Figure 6A.

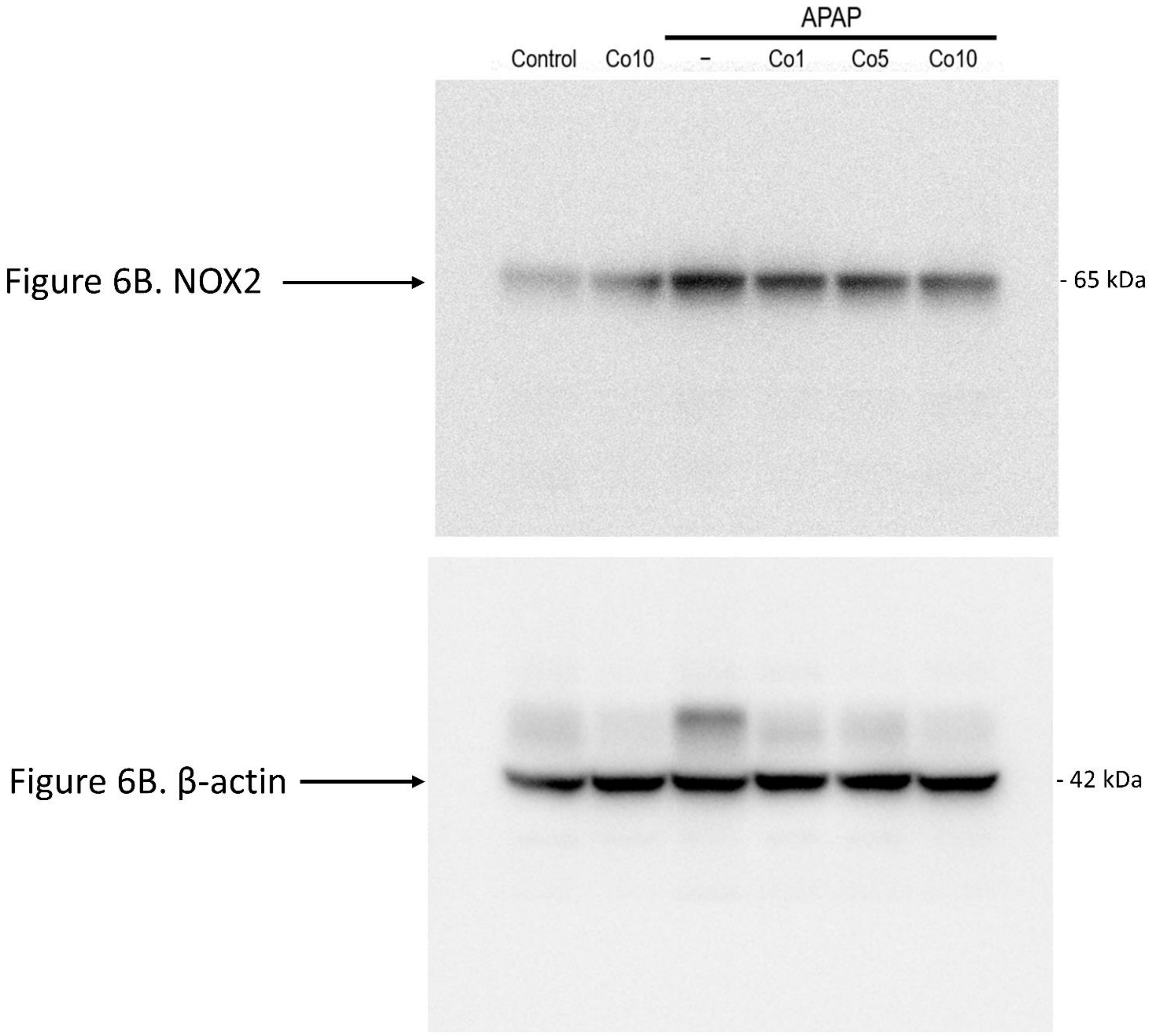

Figure S2. Uncropped Western blots from Figure 6B.

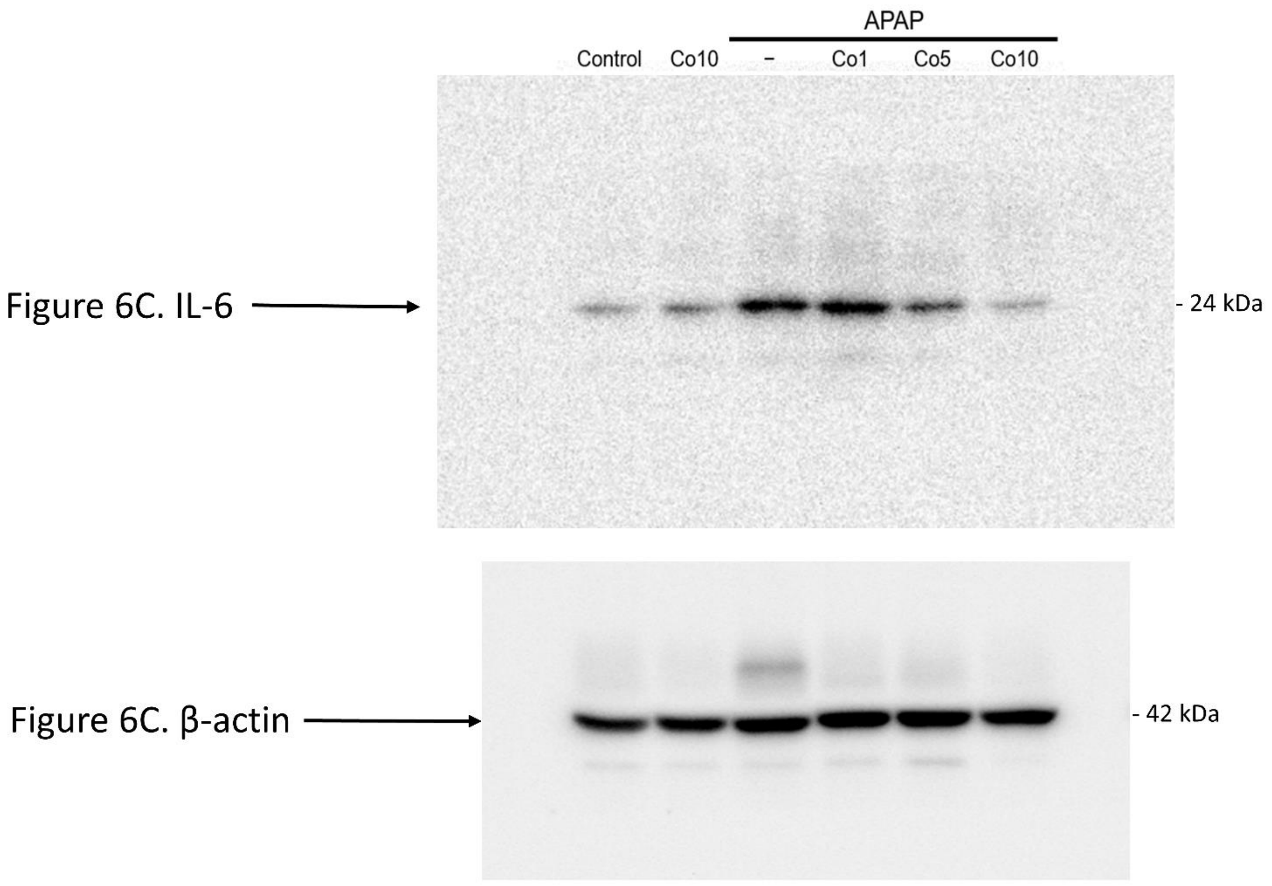

Figure S3. Uncropped Western blots from Figure 6C.

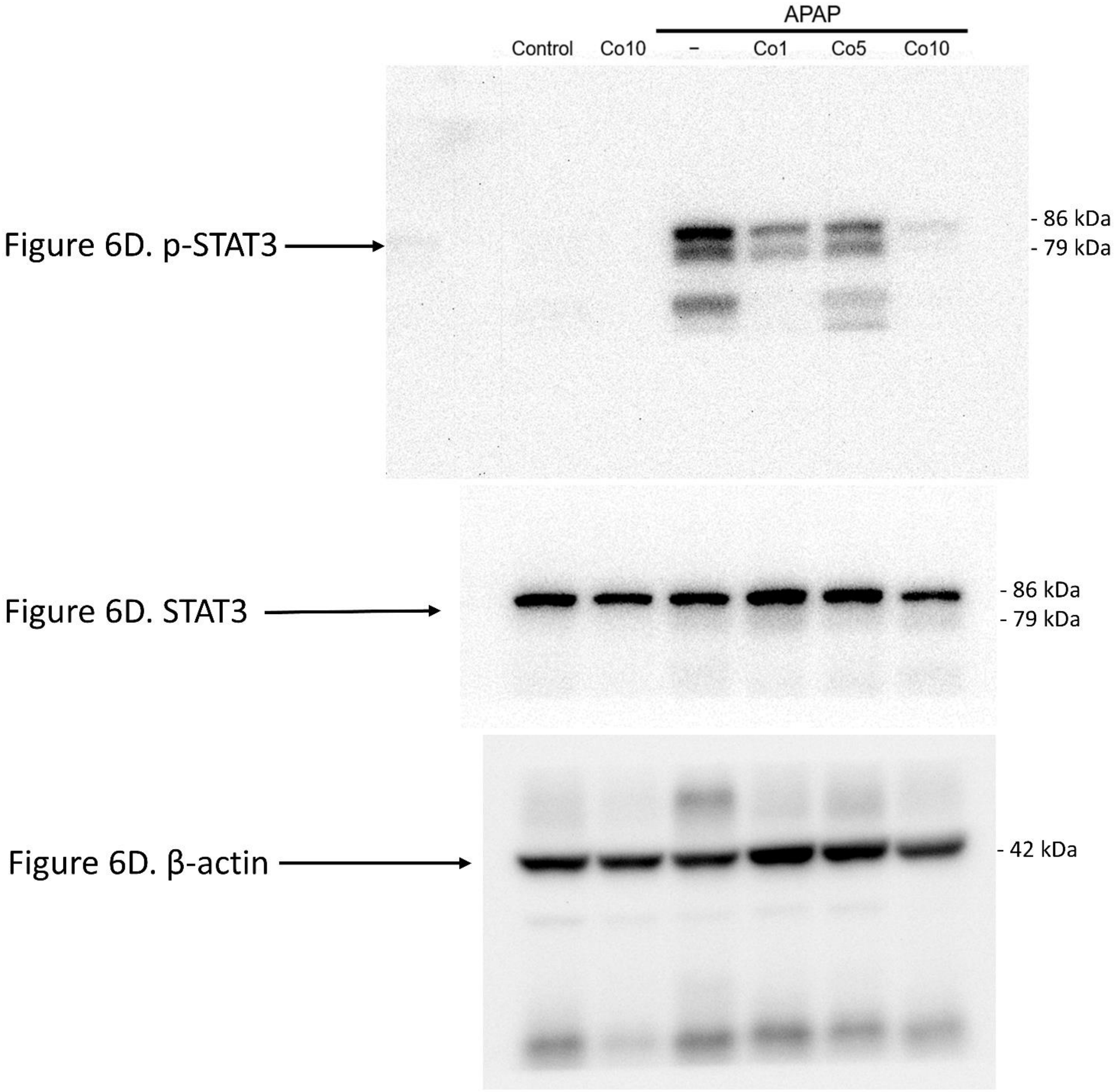

Figure S4. Uncropped Western blots from Figure 6D.

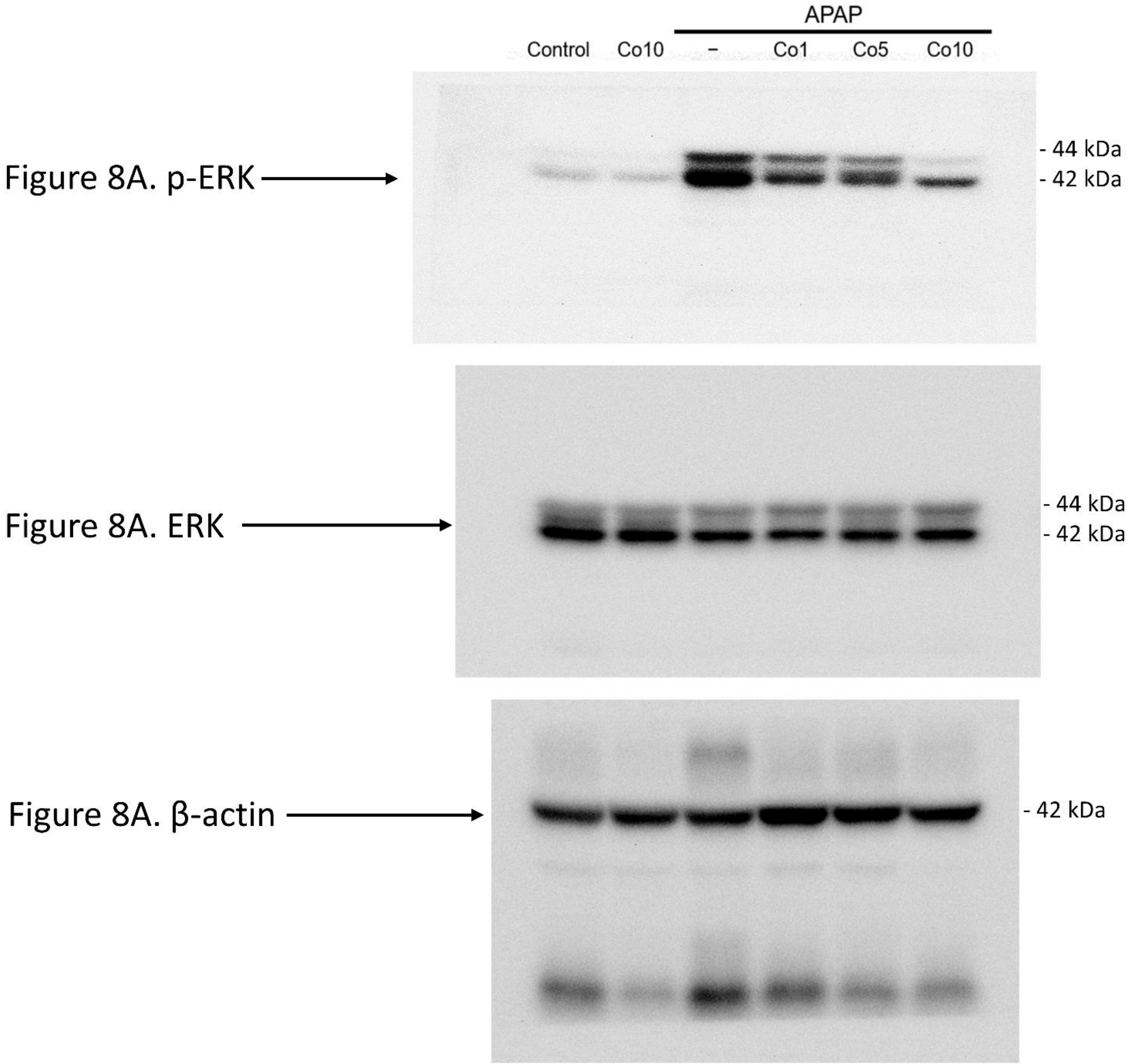

Figure S5. Uncropped Western blots from Figure 8A.

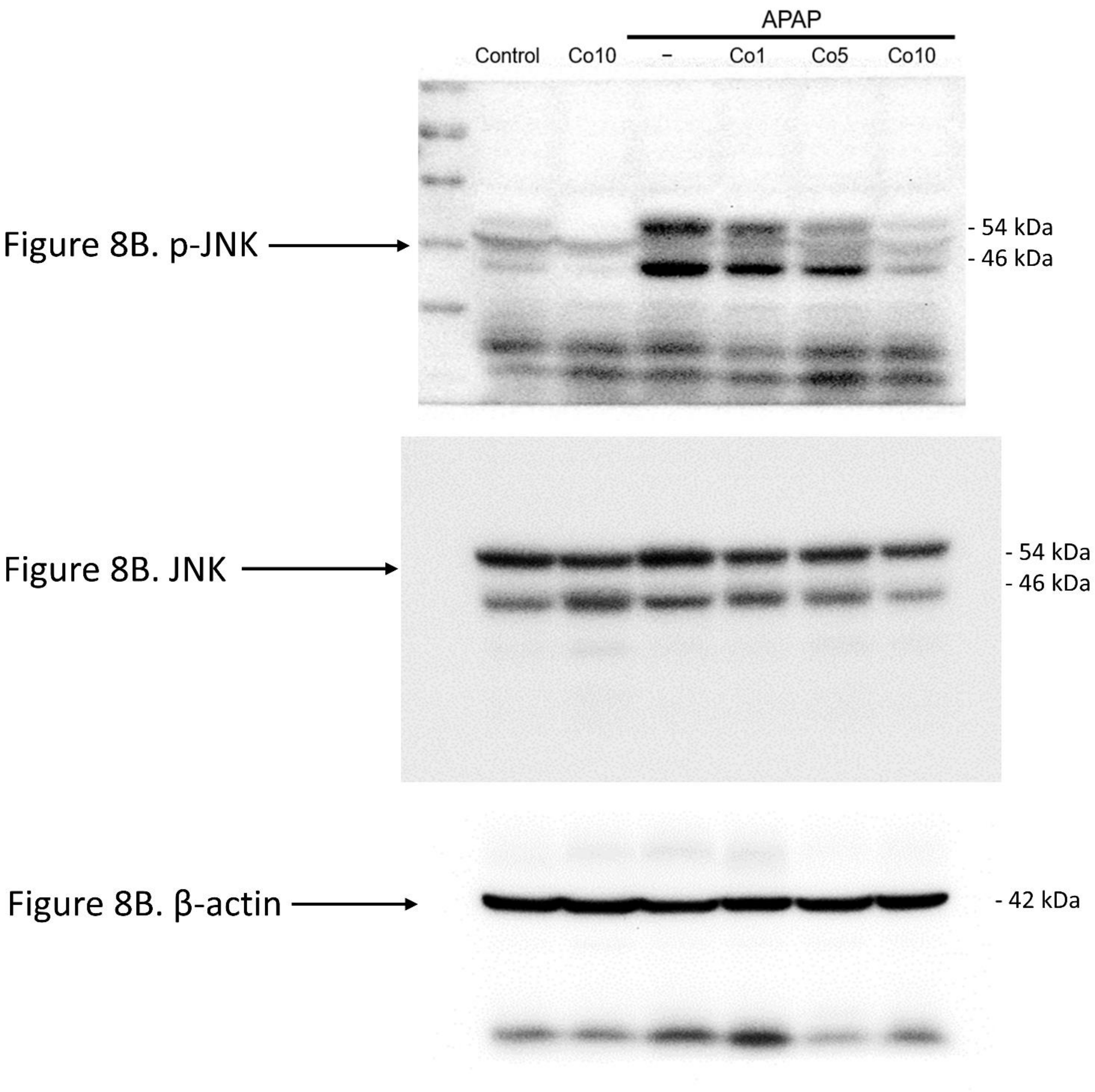

Figure S6. Uncropped Western blots from Figure 8B.

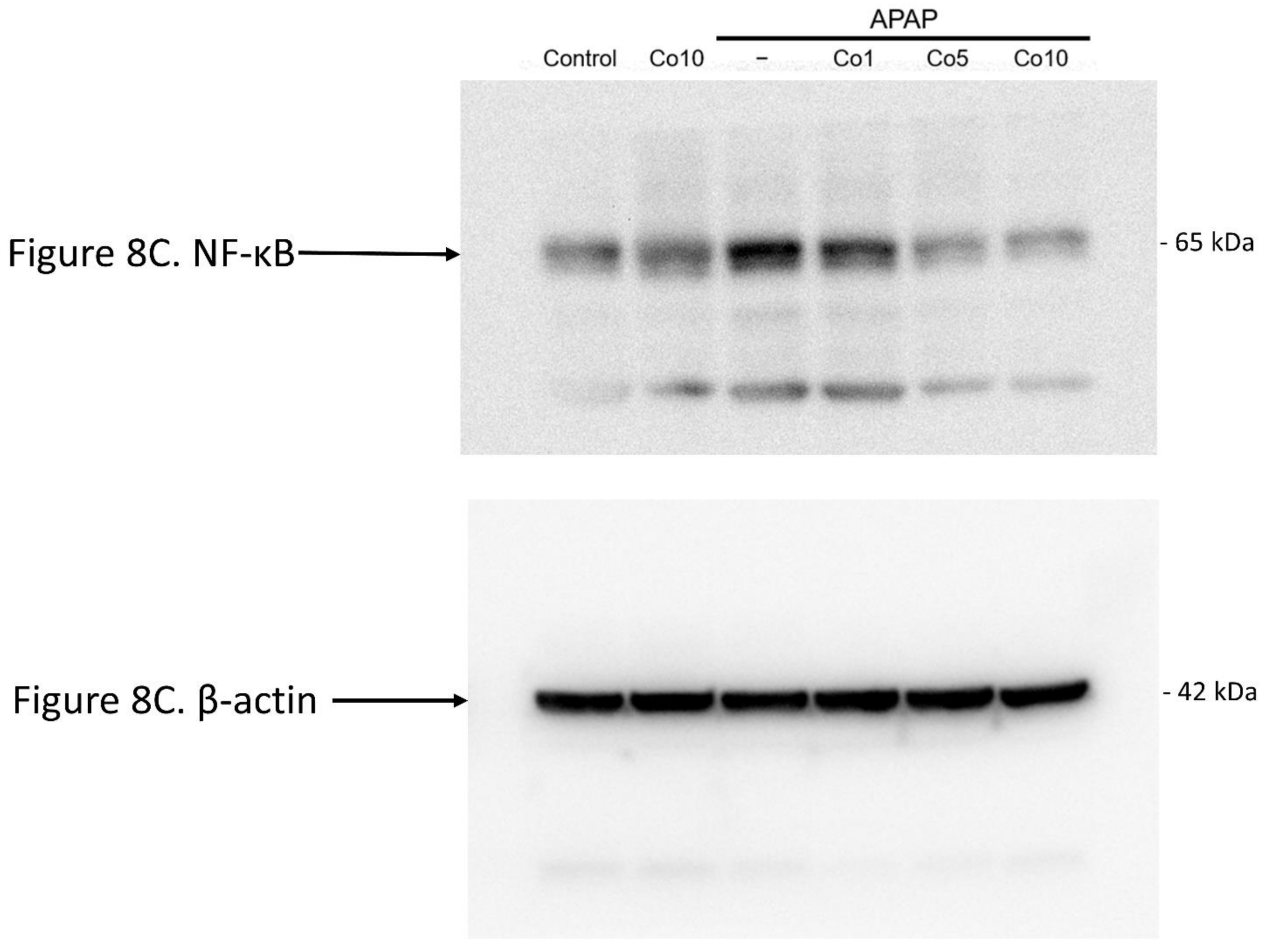

Figure S7. Uncropped Western blots from Figure 8C.
